# Structural Modeling of Djenkolic Acid with Sulfur Replaced by Selenium and Tellurium

**DOI:** 10.3390/molecules19044847

**Published:** 2014-04-17

**Authors:** Petr Melnikov, Valter A. Nascimento, Anderson F. Silva, Lourdes Z. Z. Consolo

**Affiliations:** School of Medicine of the Federal University of Mato Grosso do Sul/UFMS, Caixa Postal 549, Campo Grande/MS, Brazil; E-Mails: petr.melnikov@ufms.br (P.M.); fernandes.centrooeste@hotmail.com (A.F.S.); lzzanoni@yahoo.com.br (L.Z.Z.C.)

**Keywords:** djenkolic acid, selenium, tellurium, structure modeling

## Abstract

The comparative structural modeling of djenkolic acid and its derivatives containing selenium and tellurium in chalcogen sites (Ch = Se, Te) has provided detailed information about the bond lengths and bond angles, filling the gap in what we know about the structural characteristics of these aminoacids. The investigation using the molecular mechanics technique with good approximation confirmed the available information on X-ray refinements for the related compounds methionine and selenomethionine, as well as for an estimate made earlier for telluromethionine. It was shown that the Ch-C(3) and Ch-C(4) bond lengths grow in parallel with the increasing anionic radii. Although the distances C-C, C-O, and C-N are very similar, the geometry of conformers is quite different owing to the possibility of rotation about four carbon atoms, hence the remarkable variability observed in dihedral angles. It was shown that the compounds contain a rigid block with two Ch atoms connected through a methylene group. The standard program Gaussian 03 with graphical interface Gaussview 4.1.2 has proved to be satisfactory tool for the structural description of less-common bioactive compositions when direct X-ray results are absent.

## 1. Introduction

**Figure 1:**
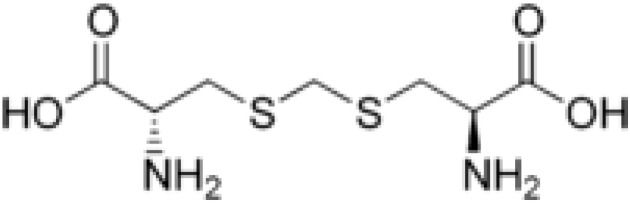
Schematicrepresentation of djenkolic acid.

Djenkolic acid (2*R*)-2-amino-3-[[(2*R*)-2-amino-3-hydroxy-3-oxopropyl]sulfanylmethylsulfanyl] propanoic acid or *S,S'*-metylenebis(cysteine), abbreviated as DjA, is an aminoacid with the structural formula shown in Figure 1.

Besides carboxylic and amino groups, it contains a pair of sulfur atoms linked to a methylene group in a way similar to that of thioeters, but different from that of sulfur atoms present in other aminoacids. It was shown to have a striking relationship to a sulfur-containing aminoacid cysteine [1]. DjA is mostly found in djenkol beans and, as stated in an earlier publication [2], severe poisoning may result from its ingestion, probably because of the neurotoxic effects of carbon disulfide. In the long run, djenkolate urolithiasis can also occur due to the precipitation of DjA in the kidneys. For a long time DjA has been provided for educational purposes only and was not intended for medical use. Recently, however, it has been mentioned as a component of skin care cosmetics and dermatological preparations acting as antioxidants [3].

It seems paradoxical, but no crystal structure of djenkolic acid has been determined by X-ray diffraction. The data on interatomic distances and angles have actually been refined only for its derivative, *S,S'*-metylenebis(cysteine) monohydrochloride [4]. Lattice parameters are available for a series of salts of djenkolic acid with transition metals [5,6]. There do not seem to have been any investigations related to the synthetic preparation of DjA analogs with S atoms replaced by other chalcogens, e.g., selenium and tellurium, although there are grounds to suppose that these closely related compounds are isostructural.

In this context, the molecular mechanics technique may be appropriate in calculating the missing parameters for comparing the structural arrangements of all three aminoacids in detail. The comparisons of the isolated models with their substituted counterparts may prove useful for our general understanding of the structure of real compounds and our ability to assess their stability and chemical properties, as well as for developing ideas for pharmacological applications.

## 2. Results and Discussion

To the best of our knowledge, the X-ray structure determination for djenkolic acid has never been performed, while the other two substances were never synthesized. As a result, the reference compounds are limited to the substituted methionine and cysteine along with their derivatives. As mentioned elsewhere [7,8], we do not perform any systematic energy sampling for searching conformational energy space. Ten conformers for each aminoacid studied were built at random for structural comparisons. Djenkolic acid and its substitution derivatives do not contain the dichalcogen bonds Ch-Ch between cysteine residues. Thus the total number of conformers compared with cysteine and cystine should be smaller due to a lack of symmetry (folding being impossible).

**Figure 2:**
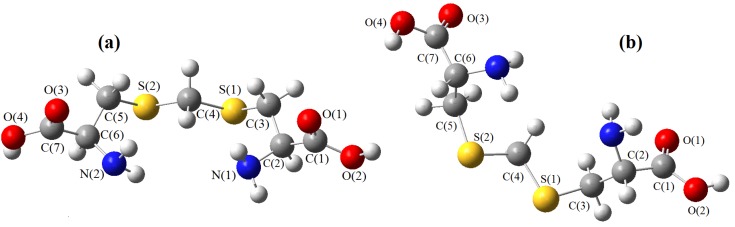
Model of djenkolic acid. (**a**) longitudinal view; (**b**) view permitting visualization at 45°.

**Figure 3:**
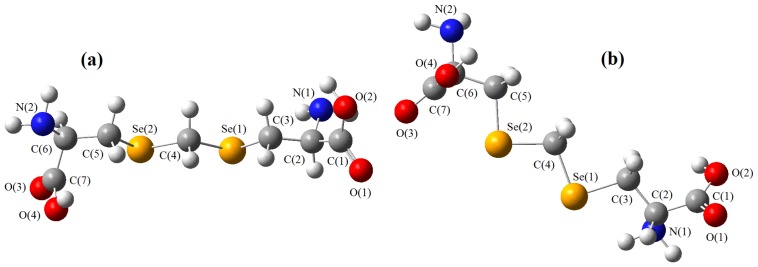
Model of djenkolic acid containing selenium. (**a**) longitudinal view; (**b**) view permitting visualization at 45°.

**Figure 4:**
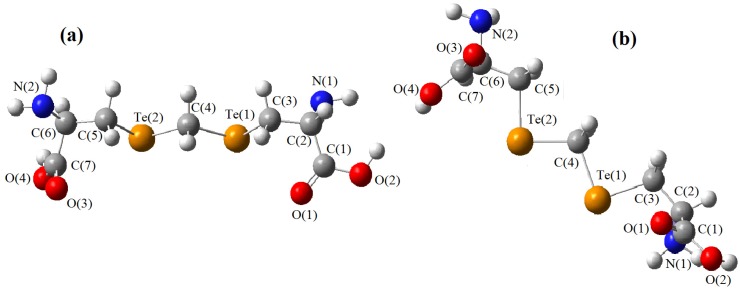
Model of djenkolic acid containing tellurium. (**a**) longitudinal view; (**b**) view permitting visualization at 45°.

The models obtained using the molecular mechanics techniques are shown in Figure 2, Figure 3 and Figure 4, where they are oriented in a comparable way. As far as homolog conformers are concerned, one can see that the chains containing chalcogens are basically the same and their geometries can be compared in further detail using the set of interatomic distances and bond angles listed in Table 1, Table 2 and Table 3.

As follows from Table 1, Table 2 and Table 3, the bond lengths C(1)-C(2), C(2)-C(3), C(5)-C(6), C(6)-C(7), are the same in the three groups of conformers presented. The typical C-C bond lengths (1.50–1.53 Å) in isolated molecules neither increase along the chain from C(1) to C(7) nor depart in a significant manner from the normal single-bond value of 1.542 Å as it occurs in the solid dl-selenomethionine [9]. All distances C-N are exactly the same (1.46 Å).

**Table 1:** Interatomic distances (Å) and angles (°), and potential energies (kJ/mol) of conformers studied.

	Conformers									
	1	2	3	4	5	6	7	8	9	10
*Distances*										
C(1)-C(2)	1.51	1.50	1.50	1.51	1.50	1.50	1.50	1.50	1.51	1.50
C(2)-C(3)	1.53	1.53	1.53	1.53	1.53	1.53	1.53	1.53	1.54	1.53
C(2)-N(1)	1.46	1.46	1.46	1.46	1.46	1.46	1.46	1.46	1.46	1.46
C(3)-S(1)	1.83	1.82	1.82	1.82	1.83	1.82	1.82	1.82	1.82	1.82
C(4)-S(1)	1.82	1.82	1.82	1.82	1.82	1.82	1.82	1.82	1.82	1.82
C(4)-S(2)	1.82	1.82	1.82	1.82	1.82	1.82	1.82	1.82	1.82	1.82
C(5)-S(2)	1.82	1.83	1.82	1.82	1.82	1.83	1.82	1.83	1.82	1.82
C(5)-C(6)	1.53	1.54	1.53	1.53	1.54	1.54	1.53	1.53	1.54	1.54
C(6)-C(7)	1.51	1.51	1.51	1.51	1.51	1.51	1.51	1.51	1.51	1.51
C(6)-N(2)	1.46	1.46	1.46	1.46	1.46	1.46	1.46	1.46	1.46	1.46
C(1)-O(1)	1.25	1.26	1.25	1.26	1.26	1.26	1.26	1.26	1.26	1.26
C(1)-O(2)	1.39	1.39	1.39	1.39	1.39	1.39	1.39	1.39	1.39	1.39
C(7)-O(3)	1.39	1.39	1.39	1.39	1.39	1.39	1.39	1.39	1.39	1.39
C(7)- O(4)	1.25	1.26	1.25	1.26	1.26	1.26	1.26	1.26	1.26	1.26
S-S	2.98	2.96	2.96	2.96	2.96	3.00	2.98	2.96	2.96	2.96
*Angles*										
C(1)-C(2)-C(3)	111.12	110.55	109.92	111.91	111.38	109.59	109.61	111.07	112.94	110.54
C(2)-C(3)-S(1)	110.69	110.50	110.97	110.57	112.35	110.89	111.04	110.45	112.10	110.89
C(3)-S(1)-C(4)	95.69	94.66	94.72	94.68	94.76	95.91	94.74	94.76	94.68	96.19
S(1)-C4)-S(2)	109.67	108.96	108.93	108.93	108.85	109.59	109.68	108.89	108.93	108.93
C(4)-S(2)-C(5)	94.71	94.71	94.74	94.73	94.77	96.91	95.66	94.78	94.68	96.17
S(2)-C(5)-C(6)	110.55	112.83	110.91	110.75	112.37	111.77	110.77	110.82	110.95	111.27
C(5)-C(6)-C(7)	110.37	113.71	111.00	111.17	111.71	112.23	111.20	111.82	113.08	110.19
O(1)-C(1)-O(2)	118.84	120.18	118.96	119.87	120.11	120.24	119.96	118.72	118.84	120.19
O(3)-C(7)-O(4)	119.88	118.64	118.95	118.83	118.76	118.72	118.03	118.76	118.72	118.82
C(1)-C(2)-N(1)	109.37	110.79	109.62	110.49	110.26	110.39	110.29	111.07	110.07	110.73
C(5)-C(6)-N(2)	110.10	111.74	110.07	110.91	111.55	111.49	110.89	111.20	109.30	111.92
C(2)-C(1)-O(1)	119.65	120.24	119.46	119.33	120.38	119.90	119.55	119.19	120.78	120.23
C(2)-C(1)-O(2)	121.50	119.56	121.56	120.79	119.47	119.85	120.49	122.07	120.67	119.57
C(6)-C(7)-O(3)	120.63	120.49	121.62	121.51	120.30	120.48	121.51	119.98	120.65	121.57
C(6)-C(7)-O(4)	119.42	120.81	119.41	119.65	120.92	120.77	119.65	119.25	120.61	119.59
*Energy* *(×10^−8^)*	−3.01	−6.19	−4.55	−2.72	−2.06	−3.27	−3.86	−4.18	−2.47	−4.75

**Table 2:** Interatomic distances (Å) and angles (°), and potential energies (kJ/mol) of conformers studied.

	Conformers									
	1	2	3	4	5	6	7	8	9	10
*Distances*										
C(1)-C(2)	1.50	1.50	1.50	1.51	1.50	1.50	1.51	1.51	1.51	1.50
C(2)-C(3)	1.53	1.53	1.53	1.53	1.53	1.53	1.53	1.53	1.54	1.54
C(2)-N(1)	1.46	1.46	1.46	1.46	1.46	1.46	1.46	1.46	1.46	1.46
C(3)-Se(1)	1.95	1.95	1.95	1.95	1.95	1.95	1.95	1.95	1.96	1.95
C(4)-Se(1)	1.94	1.94	1.94	1.94	1.94	1.94	1.94	1.94	1.94	1.94
C(4)-Se(2)	1.94	1.94	1.94	1.94	1.94	1.94	1.94	1.94	1.94	1.94
C(5)-Se(2)	1.95	1.95	1.95	1.95	1.95	1.95	1.95	1.95	1.95	1.95
C(5)-C(6)	1.53	1.53	1.53	1.53	1.53	1.53	1.53	1.53	1.53	1.53
C(6)-C(7)	1.51	1.50	1.51	1.51	1.51	1.50	1.50	1.51	1.51	1.50
C(6)-N(2)	1.46	1.46	1.46	1.46	1.46	1.46	1.46	1.46	1.46	1.46
C(1)-O(1)	1.26	1.26	1.25	1.26	1.26	1.25	1.25	1.25	1.26	1.25
C(1)-O(2)	1.39	1.39	1.39	1.39	1.39	1.39	1.39	1.39	1.39	1.39
C(7)-O(3)	1.39	1.39	1.39	1.39	1.39	1.39	1.39	1.39	1.39	1.39
C(7)-O(4)	1.26	1.26	1.25	1.26	1.26	1.26	1.26	1.25	1.25	1.26
Se-Se	3.18	3.16	3.17	3.17	3.17	3.17	3.18	3.17	3.17	3.17
*Angles*										
C(1)-C(2)-C(3)	110.40	109.54	109.98	111.95	111.36	110.17	111.15	110.08	112.87	110.18
C(2)-C(3)-Se(1)	110.69	111.23	111.13	110.68	112.85	112.24	111.02	110.63	112.40	111.44
C(3)-Se(1)-C(4)	92.32	92.44	92.25	92.27	91.21	92.30	93.12	92.25	92.21	93.63
Se(1)-C4)-Se(2)	109.61	108.94	109.14	109.06	109.15	109.06	109.59	109.14	109.10	109.11
C(4)-Se(2)-C(5)	93.09	93.63	92.17	92.31	92.20	92.30	92.97	92.27	92.18	93.63
Se(2)-C(5)-C(6)	110.99	111.64	111.22	110.94	110.98	111.93	111.24	111.04	111.07	110.93
C(5)-C(6)-C(7)	111.09	111.07	110.98	111.12	111.10	110.69	109.64	111.76	110.59	110.59
O(1)-C(1)-O(2)	119.88	119.97	118.97	119.87	120.11	118.83	118.84	118.72	118.48	118.83
O(3)-C(7)-O(4)	118.83	120.20	118.94	118.84	118.82	119.99	119.96	118.75	118.88	120.19
C(1)-C(2)-N(1)	111.60	110.59	108.62	110.48	110.29	109.26	109.40	111.09	110.09	109.21
C(5)-C(6)-N(2)	110.90	110.89	109.62	110.94	110.91	110.38	110.37	111.21	109.91	110.63
C(2)-C(1)-O(1)	119.45	120.73	119.46	119.32	120.39	119.59	119.64	119.18	120.77	119.59
C(2)-C(1)-O(2)	120.63	119.29	119.46	120.79	119.46	121.57	121.51	122.08	120.68	121.57
C(6)-C(7)-O(3)	121.51	119.92	121.55	121.50	121.52	120.42	120.46	121.98	121.49	119.57
C(6)-C(7)-O(4)	119.65	119.82	119.42	119.65	119.64	119.57	119.56	119.25	119.61	120.23
*Energy (×10^−8^)*	−3.45	−4.32	−2.73	−3.01	−2.83	−3.36	−6.96	−4.67	−4.13	−4.31

**Table 3:** Interatomic distances (Å) and angles (°), and potential energies (kJ/mol) of conformers studied.

	Conformers									
	1	2	3	4	5	6	7	8	9	10
*Distances*										
C(1)-C(2)	1.50	1.50	1.50	1.51	1.50	1.50	1.50	1.51	1.51	1.50
C(2)-C(3)	1.53	1.53	1.53	1.53	1.53	1.53	1.53	1.53	1.53	1.53
C(2)-N(1)	1.46	1.46	1.46	1.46	1.46	1.46	1.46	1.46	1.46	1.46
C(3)-Te(1)	2.15	2.15	2.15	2.15	2.15	2.15	2.15	2.15	2.15	2.15
C(4)-Te(1)	2.14	2.14	2.14	2.14	2.14	2.14	2.14	2.14	2.14	2.14
C(4)-Te(2)	2.14	2.14	2.14	2.14	2.14	2.14	2.14	2.14	2.14	2.14
C(5)-Te(2)	2.15	2.15	2.15	2.15	2.15	2.15	2.15	2.15	2.15	2.15
C(5)-C(6)	1.53	1.53	1.53	1.53	1.53	1.53	1.53	1.53	1.53	1.53
C(6)-C(7)	1.53	1.50	1.50	1.51	1.51	1.50	1.50	1.50	1.50	1.50
C(6)-N(2)	1.46	1.46	1.46	1.46	1.46	1.46	1.46	1.46	1.46	1.46
C(1)-O(1)	1.26	1.26	1.25	1.26	1.26	1.26	1.25	1.25	1.25	1.26
C(1)-O(2)	1.39	1.39	1.39	1.39	1.39	1.39	1.39	1.39	1.39	1.39
C(7)-O(3)	1.39	1.39	1.39	1.39	1.39	1.39	1.39	1.39	1.39	1.39
C(7)-O(4)	1.25	1.26	1.25	1.25	1.26	1.26	1.25	1.25	1.26	1.26
Te-Te	3.49	3.50	3.49	3.49	3.49	3.49	3.50	3.50	3.49	3.49
*Angles*										
C(1)-C(2)-C(3)	110.40	109.81	109.96	111.94	111.34	110.66	109.64	110.07	113.51	110.57
C(2)-C(3)-Te(1)	111.05	111.67	111.57	111.08	113.57	112.52	111.78	110.81	113.82	111.18
C(3)-Te(1)-C(4)	90.47	90.40	90.41	90.32	90.55	90.43	90.59	90.52	90.49	91.54
Te(1)-C4)-Te(2)	109.36	109.79	109.37	109.41	109.24	109.36	109.65	108.50	109.36	109.35
C(4)-Te(2)-C(5)	90.47	92.62	90.39	90.41	90.53	90.45	91.33	91.99	90.54	91.48
Te(2)-C(5)-C(6)	113.36	111.85	111.55	111.34	111.26	111.73	111.32	111.33	111.43	111.89
C(5)-C(6)-C(7)	111.09	111.04	110.89	111.06	111.04	110.16	111.10	111.68	110.54	110.14
O(1)-C(1)-O(2)	119.88	120.25	118.98	119.88	120.09	119.99	119.96	118.73	118.54	120.20
O(3)-C(7)-O(4)	118.84	120.19	118.95	119.82	118.83	118.83	118.83	118.75	118.89	118.83
C(1)-C(2)-N(1)	111.62	109.56	109.62	110.51	110.27	110.78	110.23	111.15	111.23	110.78
C(5)-C(6)-N(2)	110.92	110.92	110.07	110.91	110.97	111.73	110.92	111.20	109.94	111.88
C(2)-C(1)-O(1)	119.45	120.11	119.46	119.33	120.40	119.58	119.56	119.18	120.70	120.23
C(2)-C(1)-O(2)	120.64	119.62	121.54	120.78	119.46	120.42	120.46	122.07	120.69	119.56
C(6)-C(7)-O(3)	119.65	119.87	121.60	121.51	121.51	121.57	121.50	119.25	119.61	119.58
C(6)-C(7)-O(4)	121.50	199.92	119.42	119.66	119.65	119.59	119.65	121.98	121.48	121.57
*Energy (×10^−8^)*	−2.09	−3.52	−2.66	−4.08	−3.73	−2.95	−3.50	−3.08	−2.03	−3.63

The main crystallographic data obtained for methionine and selenomethionine so far comprise the crystal structure of d,l-alanylmethionine [10], the crystal structure of the α and β forms of d,l-methionine [11] and the crystal structure of d,l-selenomethionine [9], as well as the crystal and molecular structure of d,l-methionine nitrate [12]. The latest contribution to this topic is a report on the structure and dynamics of l-selenomethionine in the solid state [13] which successfully combines X-ray studies and solid state NMR spectroscopy.

**Figure 5:**
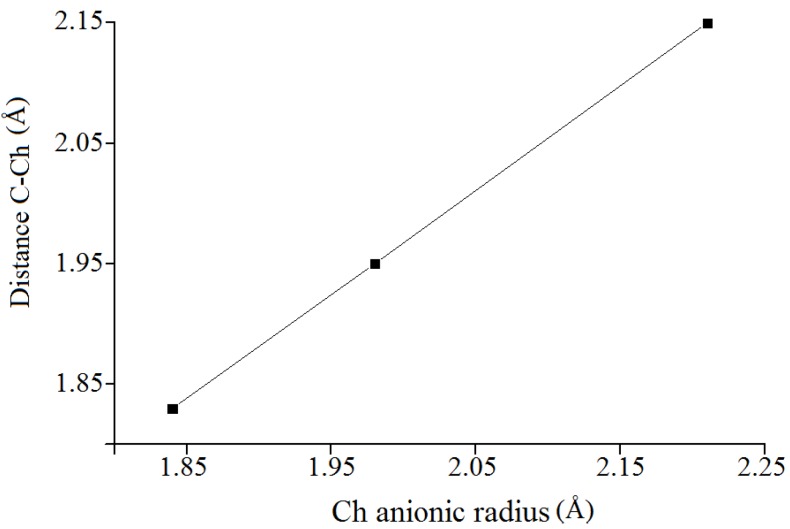
Dependence of the distances C-Ch on the chalcogen anionic radii.

As expected, the distances C(3)-Ch(1), C(4)-Ch(1), C(4)-Ch(2), and C(5)-Ch(2) are in the same range within the groups of conformers but different for each chalcogen: C-S = 1.82 Å; C-Se = 1.94–1.95 Å; and C-Te = 2.14–2.15 Å. In DjA all C-S distances are in accordance with the average value 1.807 Å computed for 546 substances containing this fragment in the Cambridge Structural Database [14]. They are also in accordance with later published sources [8,10,11]. As for the same distances in DjSe, they are identical with those reported on the basis of X-ray studies (1.899–1.97 Å) [9,13]. The X-ray data for DjTe are missing in literature, so we compared our value with data for the C-Te distance characteristic of tellurium-containing organic compounds, which is 2.158 Å [15] exactly the same as found in our present research, and as estimated for telluromethionine earlier [8]. When plotted *vs* the chalcogen anionic radii (Figure 5) the bond lengths C-Ch showed a net linear dependence on this parameter. As can be seen, the C-Te distances are much larger than those of the C-Se and C-S bonds. This seems to indicate that the cleavage of the central ditelluride unity would occur easier, and as a result pharmacological properties may be impaired.

The angles C-Ch-C are smaller than the average value of 98.75°, as published in the Cambridge Structural Database [14], and closer to those of substituted methionines (94.62–96.18°) [8]. They decrease as follows: 95.08° for DjA, 92.4° for DjSe and 90.57° for DjTe. Naturally, this contraction would lead to increased distances between chalcogen atoms, a fact which is actually confirmed by the corresponding calculated values: 2.96–3.00 Å for DjA, 3.16–3.18 Å for DjSe, and 3.49–3.50 Å for DjTe. It must also be borne in mind that the angles C(1)-C(2)-C(3) do not show any substantial scattering in the three compounds studied. By consequence, this indicates that a rigid structural block 
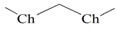
 must be located in the same plane. This condition limits the number of possible conformers because of hindered rotation about C(4), so most of the rotamers must be generated in the peripheral areas. It is interesting that the aforementioned blocks are reminiscent of the fragments S····S belonging R-type ring motifs, characteristic for the structure of l-cysteines obtained at high pressures [16]. The main effects of compression were to reduce S····S distances from 3.846 to 3.450 Å. In our case of two cysteine molecules linked through a methylene group these distances are even shorter: 2.96–3.50 Å, coinciding only at the upper limit.

As for the remaining angles of the main chain C(1)-C(2)-C(3) and C(5)-C(6)-C(7), they do not appear to be sensitive to the chalcogen nature, being within a range of 110.77–111.65° for all models considered in this study.

The isolated nonionized forms of djenkolic acid and its derivatives are intrinsically flexible systems, so they are expected to display the largest variety of torsion (dihedral) angles. As has been observed, their coincidences are not so precise, although the averages can be roughly subdivided into three groups: ±70°, ±109°, and ±172°. With the exception of the angle comprising the amino group, these values are close to those published for d,l-selenomethionine and substituted methionines [8,10].

The energy barriers that separate different conformers are typically rather small for many conformations so that the thermal energy at room temperature enables the molecule to freely change from one conformation to another [17]. The values of minimal potential energies are given at the bottom of Table 1, Table 2 and Table 3. They are of the same narrow order (−6.99–−2.03), meaning that the conformers have comparable stability for sulfur, selenium and tellurium. It is clear that these levels, calculated by means of molecular mechanics do not necessarily have any physical meaning in themselves and are not comparable between different force fields. However, when considering several structures closely related, the method appears to be extremely useful.

## 3. Material and Methods

In this work, the structures resulting from substitution of oxygen and tellurium for sulfur in methionine were simulated using the universal force field (UFF) potential as implemented in the Gaussian 03 [18] and Gaussview 4.1.2 [19] software packages. The “force field” is a set of algebraic expressions, which describes how changes in the bond length, bond angles, torsion angles, etc. affect the energy of a particular structure. Since different force fields are constructed on the basis of different empirical data, they may emphasize different terms describing a particular class of molecules.

UFF is a force field widely used for systems containing elements other than carbon, hydrogen oxygen and nitrogen. It contains the energy terms for bond stretching, angle bending, dihedral torsion, Van der Waals and electrostatic potential [20,21]. Previously, we also tried to employ the GHEMICAL 2.98 package with a Trypos 5.2 force field program and the HYPERCHEM 7.5 software package with a MM + force field program, but they showed themselves to be weaker instruments [7], most probably due to the absence of a complete set of parameters for the chalcogens beyond sulfur. *In vacuo* calculations would bring out most of the underlying conformations without being side-tracked by the solvent used in the study or the limitations imposed by the densest packing. Strictly speaking, no conformational search routine guarantees that all conformers have been found, so the strategy chosen in this work was to study a reasonably representative set of the optimized geometries. The geometry optimization was carried out in Cartesian coordinates using the Berny optimization algorithm, adjusting the parameters until a stationary point on the potential surface was found. That means that for a small displacement the energy does not change within a certain amount, and the placements are successfully converged. Angles and interatomic distances were evaluated by using special features of the program.

## 4. Conclusions

The comparative structural modeling of djenkolic acid and its derivative containing selenium and tellurium in chalcogen sites (Ch = Se, Te) has provided detailed information about the bond lengths and bond angles, filling the gap in the structural characteristics of these aminoacids. The investigation using the molecular mechanics technique with good approximation confirmed the available information on X-ray refinements for the related compounds methionine and selenomethionine, as well as for estimates made earlier for telluromethionine. It was shown that Ch-C(3) and Ch-C(4) bond lengths grow in parallel with the increasing anionic radii. Although the C-C, C-O, and C-N distances are very similar, the geometry of conformers is quite different due to the possibility of rotation about four carbon atoms, hence the remarkable variability in dihedral angles. It was shown that the compounds contain a rigid block containing two Ch atoms connected through a methylene group. The standard program Gaussian 03 with graphical interface Gaussview 4.1.2 has proved to be satisfactory for the structural description of less-common bioactive compositions when direct X-ray results are missing.
